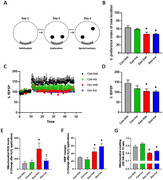# Hyperpolarization‐activated cyclic nucleotide‐gated channel inhibitor, Ivabradine, attenuates brain mitochondrial oxidative stress without reducing cognitive impairment in Dox‐induced chemobrain

**DOI:** 10.1002/alz.085164

**Published:** 2025-01-03

**Authors:** Hiranya Pintana, Nattayaporn Apaijai, Adivitch Sripusanapan, Chotrawee Piriyakunthorn, Titikorn Chunchai, Damrongsak Jinarat, Busarin Arunsak, Nipon Chattipakorn, Siriporn C Chattipakorn

**Affiliations:** ^1^ Neurophysiology Unit, Cardiac Electrophysiology Research and Training Center, Faculty of Medicine, Chiang Mai University, Chiang Mai Thailand; ^2^ Center of Excellence in Cardiac Electrophysiology Research, Chiang Mai University, Chiang Mai Thailand; ^3^ Office of Research Administration, Chiang Mai University, Chiang Mai Thailand; ^4^ Department of Physiology, Faculty of Medicine, Chiang Mai University, Chiang Mai Thailand; ^5^ Department of Oral Biology and Diagnostic Sciences, Faculty of Dentistry, Chiang Mai University, Chiang Mai Thailand

## Abstract

**Background:**

Doxorubicin (Dox), a chemotherapeutic agent, is known to cause chemobrain leading to cognitive decline and brain mitochondrial dysfunction. Ivabradine (Iva), hyperpolarization‐activated cyclic nucleotide‐gated channel blocker used for angina and arrhythmia, has been shown to be an anticonvulsant, antioxidant, and neuroprotective agent. However, the effects of Iva on cognitive function, and brain mitochondrial function in Dox‐induced chemobrain are still not determined. This study aimed to investigate whether Iva attenuates Dox‐induced brain toxicity in male rats by affecting cognitive dysfunction, synaptic dysplasticity, and brain mitochondrial dysfunction.

**Method:**

Twenty male Wistar rats were divided into 4 groups: vehicle‐treated control, Iva‐treated control, vehicle‐treated Dox, and Iva‐treated Dox. The control rats were received normal saline, while the Dox‐treated rats were received Dox at a dose of 3 mg/kg/day intraperitoneally on days 0, 4, 8, 15, 22, and 29. Iva (10 mg/kg) was administered by oral gavage from day 0 to day 29. On day 30, novel object location (NOL) test was performed to assess cognitive function. Hippocampal‐synaptic plasticity and brain mitochondrial function were measured after euthanasia.

**Result:**

Dox‐treated rats showed a decreased % preference index of a new location in the NOL test, decreased % of field excitatory postsynaptic potential (fEPSP), and impaired brain mitochondrial function as indicated by increased mitochondrial ROS, impaired brain mitochondrial membrane potential change, and caused mitochondrial swelling **(Figure 1A‐G)**. Iva‐treated control rats showed no effects on the NOL, % fEPSP, and brain mitochondrial function compared to vehicle‐treated control rats. Iva‐treated Dox rats exhibited a decrease in brain mitochondrial reactive oxygen species (ROS), but showed no significant difference in NOL, % fEPSP, brain mitochondrial membrane potential change, and mitochondrial swelling compared to vehicle‐treated Dox rats **(Figure 1A‐G)**.

**Conclusion:**

Dox‐induced chemobrain manifested in cognitive impairment, synaptic dysplasticity, and brain mitochondrial dysfunction. Iva treatment showed a reduction in brain mitochondrial ROS, with no improvement in mitochondrial membrane potential changes and swelling in Dox‐induced chemobrain. Iva did not improve cognitive function and hippocampal‐synaptic plasticity in Dox‐treated rats. These findings suggest the antioxidant properties of Ivabradine in chemobrain, but this dose of Iva was not enough to protect brain injury in Dox‐induced chemobrain.